# The Complexities of Navigating the Healthcare System as an Autistic Individual with Ehlers-Danlos Syndrome: A Patient Perspective

**DOI:** 10.1177/23743735251333601

**Published:** 2025-04-15

**Authors:** Sarah Clark

**Affiliations:** 6657 Bournemouth University, Poole, UK

**Keywords:** access to care, caregiving, clinician-patient relationship, communication, diversity, education, empathy, equity

## Abstract

Autistic individuals with hypermobile Ehlers-Danlos syndrome (hEDS) often face unique challenges navigating healthcare systems due to lack of clinician awareness, diagnostic delays, misdiagnoses, and systemic barriers. Drawing on my own lived experience within the UK's National Health Service (NHS), this patient perspective article integrates personal narratives with the research and literature, highlighting critical gaps in clinical care, offering practical recommendations to enhance equity and inclusion. *Recommendations include:* improved clinician education on neurodivergence and overlapping conditions, improving holistic patient-centred communication, and collaborative care models that prioritise patient needs, learning from lived expertise.

## Introduction

Patients with co-occurring autism and hypermobile Ehlers-Danlos syndrome (hEDS) often face significant barriers to accessing equitable and effective healthcare.^1,2^ Both conditions are often overlooked and misunderstood, leading to significant delays in diagnosis and misdiagnoses that exacerbate health inequities. Additionally “The Tripple Empathy Problem” further illustrates possible communications challenges in healthcare settings for autistic people.^3^

As a late-diagnosed autistic individual diagnosed with hEDS after years of misdiagnoses and inadequate care, I have first-hand experience with these challenges.^4^ This perspective amplifies the voices of patients like me, who encounter systemic barriers and the emotional toll of seeking and appropriate care in healthcare.

Any recommendations made here are, of course, given with full recognition of the challenges medical staff face, including: high stress, time constraints, and limited and highly stretched resources. I also sincerely appreciate the efforts of all the healthcare professionals who have tried to support me over the past twenty or so years, even though a lack of appropriate awareness and knowledge often led to misdiagnosis, inappropriate clinical pathways, over-medication, ineffective and even harmful treatments.

## Background

hEDS and autism frequently co-occur, suggesting a connection beyond chance.^5^ Increasing evidence highlights a significant overlap, with research exploring potential shared genetic links. hEDS is the most common of the Ehlers-Danlos syndromes, a group of genetic connective tissue disorders that affect multiple bodily systems due to defects in collagen and related connective tissues. Key symptoms often include joint hypermobility, chronic pain, fatigue, dizziness, easy bruising, and gastrointestinal or bladder dysfunction. Despite its widespread effects, hEDS remains significantly underdiagnosed and poorly understood.

Autism is a lifelong neurodevelopmental condition, and when viewed through the medical model is characterised by: differences in social communication, sensory processing, repetitive behaviours, and an often an increased likelihood of co-occurring mental health challenges. However, through the social model of disability, autism is seen as a *difference* (with many strengths) rather than a deficit. Rather than autism itself being disabling, societal barriers are seen to create challenges. However, with inclusive environments, reasonable adjustments (including in healthcare settings) and improved understanding and awareness, autistic individuals can thrive in the right environments.

Autistic individuals with hEDS often experience overlapping symptoms, including sensory sensitivities, altered pain perception, difficulties with emotional regulation, sleep disturbances, coordination challenges, and cognitive differences such as difficulties with concentration and memory. A 2016 Swedish study^6^ estimated that nearly 8% of autistic individuals also have Ehlers-Danlos syndrome. However, the true prevalence remains unclear due to diagnostic barriers and significant underdiagnosis. Recognizing the relationship between autism and hEDS is crucial for improving clinical awareness, earlier diagnosis, and more appropriate care and support.

Autistic individuals with hEDS or hypermobility spectrum disorder (HSD) can often face complex and multifaceted challenges that significantly impact their health and well-being. Many individuals often endure lengthy and distressing diagnostic journeys, frequently marked by clinician-associated traumatisation and medical invalidation. Such experiences can have profound psychological consequences, contributing to distress, hopelessness, and, in some cases, suicidality. Furthermore, misdiagnoses and delays in recognition often result in poorer health outcomes, physical de-conditioning, and exposure to inappropriate or even harmful treatments.

## Patient Perspectives

### Diagnostic Delays and Misdiagnoses

My own diagnostic journey spanned over 15 years of misdiagnoses, including misdiagnosed psychiatric labels such as Emotionally unstable personality disorder (EUPD) and neurotic depression. These misdiagnoses undermined my credibility and led to underlying physical medical conditions being overlooked and not believed. Tamilson et al highlight experiences similar to mine of late-diagnosed autistic females, previously misdiagnosed with EUPD.^7^

Many years of medical invalidation and gaslighting took a severe psychological toll on my wellbeing and also resulted in me becoming physically sicker and sicker. Research corroborates that autistic individuals are at higher risk of being misdiagnosed with mental health conditions, often overshadowing physical health concerns.^8^ Studies show that the average time to diagnosis for hEDS can exceed a decade, with patients frequently dismissed due to the *perceived* rarity of the condition and lack of clinician awareness.^9^

Receiving a diagnosis of hEDS in 2020, and later autism in 2022, provided a framework to start understanding my symptoms (EDS) and communication and social differences (autism) and allowing me start to advocate for more appropriate healthcare. However, these delays had already led to significant physical and emotional harm. The intersectionality of autism and hEDS demands greater awareness among clinicians to avoid perpetuating cycles of misdiagnosis and patient harm.

### Healthcare System Barriers

Navigating healthcare systems as an autistic person presents unique challenges, such as sensory sensitivities in healthcare environments, the potential for greater difficulty with verbal communication during stress, and the need for clear, direct information, which is often unmet in traditional care settings. Additionally, the lack of coordinated care for co-occurring conditions such as Postural orthostatic tachycardia syndrome (POTS) and Mast cell activation syndrome (MCAS) further fragmented my patient experience.

The healthcare environment itself often exacerbates stress for autistic individuals. Bright lights, long waiting times, and the unpredictability of appointments can be overwhelming. These factors highlight the urgent need for trauma-informed, neuro-affirmative practices in healthcare. Additionally, the pressures faced by medical staff, including high patient loads, limited resources, and time constraints, should be acknowledged to foster mutual understanding and collaboration.

### The Emotional Toll

The process of advocating for oneself within a pressured and hierarchical healthcare system that frequently invalidates patient experiences is emotionally exhausting. Due to possible alexithymia, interoceptive and proprioceptive differences, autistic individuals may struggle to be able to accurately describe pain or advocate effectively in high-pressure medical scenarios. This can lead to feelings of helplessness and frustration. Research indicates that medical trauma is a significant issue for individuals with complex conditions who face repeated medical dismissal or clinician-associated traumatisation.^10^ My own experiences of being misunderstood and dismissed have fueled my commitment to amplifying patient voices and pushing for systemic change.

## Practical Recommendations

1. Clinician Education and Training:
  - Provide mandatory training on autism and co-occurring physical health conditions (such as EDS) for healthcare professionals, emphasising their intersection.
  - Include patient perspectives and the lived experience voice in training materials to foster greater empathy and understanding.
2. Patient-Centered Communication:
  - Use accessible language (eg, avoiding medical jargon, and allowing autistic patients additional time to process and respond to questions due to executive functioning differences) and visual aids to explain medical concepts.
  - Offer alternative communication methods, such as written or visual tools, during appointments.
3. Trauma-Informed Care:
  - Create sensory-friendly healthcare environments (eg, dimmable lights, quiet waiting areas).
  - Ensure clinicians are trained to recognise signs of neurodivergence and promote training on neuro-affirmative and experience-sensitive care.
  - Ensure clinicians are trained to recognise signs of medical trauma and respond compassionately.
4. Integrated Care Pathways:
  - Develop multidisciplinary care teams to address the complex needs of patients with co-occurring conditions.
  - Streamline referral processes to reduce delays and improve continuity of care to ultimately reduce unnecessary delays, implementing direct referral systems between specialists, and ensuring that referrals are directed to appropriate services without excessive administrative barriers.
5. Patient Advocacy and Support:
  - Encourage peer-support networks to empower patients and reduce feelings of isolation.
  - Provide guidance for autistic individuals on self-advocacy strategies, including preparing written summaries for medical appointments, and provide training for clinicians to allow for written information from patients where appropriate if it better aids in doctor-patient communication.
6. Advocacy Within the NHS:
  - Promote lived-experience-led policy discussions to drive autism-inclusive healthcare improvements.

## Conclusion

The overlap and co-occurrence of autism and hEDS presents a number of unique challenges that demand systemic changes in healthcare. By integrating patient perspectives, changing and improving clinician education, and fostering inclusive trauma-informed and neuro-affirmative healthcare environments, we can begin to address the inequities faced by this population. My own patient narrative and diagnostic journey underscores the importance of listening to patient voices to inform meaningful improvements in healthcare delivery. Moving forward, a commitment to equity, inclusion, and patient-centred care can transform healthcare experiences and outcomes for those navigating these intersecting conditions. Ultimately this should improve health outcomes, reduce mortality rates, and improve the psychological well-being of this patient group as well as potentially helping to reduce the high suicide rate among this population.
